# The California collaborative network to promote data driven care and improve outcomes in early psychosis (EPI-CAL) project: rationale, background, design and methodology

**DOI:** 10.1186/s12888-024-06245-6

**Published:** 2024-11-14

**Authors:** Valerie L. Tryon, Kathleen E. Nye, Mark Savill, Rachel Loewy, Madison J. Miles, Laura M. Tully, Andrew J. Padovani, Daniel J. Tancredi, Joy Melnikow, Sabrina Ereshefsky, Nitasha Sharma, Amanda P. McNamara, Merissa Kado-Walton, Christopher Komei Hakusui, Chelyah Miller, Khanh Linh H. Nguyen, Maliha Safdar, Viviana E. Padilla, Leigh Smith, Adam B. Wilcox, Lindsay M. Banks, Stephania L. Hayes, Katherine M. Pierce, Karina Muro, Daniel I. Shapiro, Khalima A. Bolden-Thompson, Renata M. Botello, Rebecca E. Grattan, Yi Zhang, Bonita Hotz, Lisa Dixon, Cameron S. Carter, Tara A. Niendam

**Affiliations:** 1 University of California Davis Medical Center, Sacramento, CA USA; 2 University of California, San Francisco, CA USA; 3 University of California, San Diego, CA USA; 4 Washington University in St. Louis, St. Louis, MO USA; 5 Case Western Reserve University, Cleveland, OH USA; 6 Boston University, Boston, MA USA; 7 Victoria University of Wellington, Wellington, New Zealand; 8 New York State Psychiatric Institute, New York, NY USA; 9 Columbia University, New York, NY USA

**Keywords:** Early psychosis, Coordinated specialty care, Learning health care network, EPINET

## Abstract

**Background:**

A prolonged first episode of psychosis (FEP) without adequate treatment is a predictor of poor clinical, functional, and health outcomes and significant economic burden. Team-based “coordinated specialty care” (CSC) for early psychosis (EP) has established effectiveness in promoting clinical and functional recovery. However, California’s CSC program implementation has been unsystematic and could benefit from standardizing its processes and data collection infrastructure. To address this, we established a consortium of EP clinics across the state via a Learning Health Care Network (LHCN) framework to develop the Early Psychosis Intervention Network of California (EPI-CAL). EPI-CAL’s LHCN developed a core battery of evidence-based measures for service users and family members and linked them together using a unique data collection and visualization application, Beehive.

**Methods and objectives:**

EPI-CAL’s LHCN collects, visualizes, and aggregates data at the individual and clinic level for EP programs across California via Beehive. Beehive was designed to: (1) collect outcomes data from service users receiving care at EP programs and their support persons, (2) provide the data to providers on a secure web-based dashboard to support measurement-based care, and (3) allow data to be used for program or research analysis. We will (1) determine the feasibility of implementing an LHCN across a diverse, decentralized network of early psychosis programs, (2) determine if the implementation of an LHCN increases the delivery of measurement-based care, and (3) determine if the implementation of measurement-based care is associated with significant improvements in key service user outcomes. EPI-CAL’s network will contribute data to the Early Psychosis Intervention Network (EPINET) program.

**Discussion:**

The current study aims to establish an LHCN of EP clinics in California that implements harmonized data collection using Beehive and assesses the feasibility of establishing such a network. Our goal is for this harmonized data collection approach to be used to inform decisions and develop learning opportunities for service users, staff, and administrators, and to improve outcomes for service users and their supporters in CSC care. Further, the data will enable programs and research teams to examine what elements of care lead to program success and improved treatment outcomes for service users.

**Clinical trials registration:**

www.ClinicalTrials.gov, identifier NCT04007510; registered 07/05/2019.

**Supplementary Information:**

The online version contains supplementary material available at 10.1186/s12888-024-06245-6.

## Introduction

A prolonged first episode of psychosis (FEP) without adequate treatment is a consistent predictor of poor clinical and functional outcomes [1], poor health outcomes [2], and significant economic burden [3]. Individuals with psychotic disorders can experience positive symptoms (e.g., delusions and hallucinations), negative symptoms (e.g., reduced motivation, difficulty expressing emotions) [4], and cognitive impairments that may significantly impact daily functioning [1].

Team-based “coordinated specialty care” (CSC) [5] for early psychosis (EP) has established effectiveness in promoting clinical and functional recovery [6]. This intervention includes case management and coordination, ongoing psychiatric and/or medical assessments and treatment, service user and support person psychoeducation and psychotherapy, educational and vocational support, and relapse prevention. Recent state and federal initiatives have led to the rapid and widespread dissemination of CSC across the United States, such as New York [7] and Texas [8]. However, CSC program development has been disconnected both across and within states and specific clinical services offered can vary as a result. For example, CSC programs in California have developed locally within specific health systems and counties and with little coordination with each other. This lack of state and national coordination and data infrastructure limits the capacity for data-based innovation, accelerated dissemination of best practices, and broad-scale evaluation [9]. To address this, EPI-CAL proposed to develop a learning health care network (LHCN) of EP programs across California.

The National Academy of Sciences defines an LHCN as a system in which “science, informatics, incentives, and culture are aligned for continuous improvement and innovation, with best practices seamlessly embedded in the delivery process, patients and families are active participants in all elements, and new knowledge captured as an integral by-product of the delivery experience” [10]. In other health areas, LHCNs have been found to lead to a multitude of benefits, including better adherence to evidence-based best practices [11], improved understanding of service user experiences [12], increased remission rates [13], shorter wait times [11], and high levels of service user satisfaction [14]. However, measurement-based health care, a key feature of an LHCN, is not currently standard practice in US mental health care [15]. Systems that have implemented LHCNs in early psychosis, such as OnTrackNY’s learning health care system, have been made achievable through a centralized approach to program development, implementation, training, and program evaluation of a single CSC model for multiple programs (OnTrackNY) [7, 16]. A key feature of this project will be determining the feasibility of implementing an LHCN in a decentralized system, across diverse independent clinics adopting different approaches to CSC care delivery [17]. If feasible, the findings could provide a framework to support the broader implementation of LHCNs in the mental health care setting.

EPI-CAL’s LHCN establishes a network incorporating existing California EP clinics based in both community and university settings. Programs within the network all collect a core battery of evidence-based measures for service users, support persons, and care providers using a unique data collection and visualization application, Beehive. These data are then de-identified and made accessible to the central research team to support quality improvement research. EPI-CAL is also part of NIMH’s national Early Psychosis Intervention Network (EPINET); EPINET is a national learning health care system for early psychosis that seeks to coordinate a series of early psychosis networks at the national level. EPI-CAL provides key support, valuable innovation, and diversity to the EPINET initiative. EPI-CAL makes a change to existing practice in the field of mental health by implementing a collaborative LHCN, supporting quality improvements, service user engagement, and provider use of measurement-based care in EP programs. This LHCN collects, visualizes, and aggregates real-time data at the individual and clinic level to inform service user-, program-, county-, and state-level decisions and develop learning opportunities for individuals, staff, programs, and administrators to improve service user outcomes.

The best method for harmonizing core data metrics and outcome measures across diverse community- and university-based EP programs has not been established. Providers need sufficient motivation, training, and support to implement measurement-based care in treatment sessions and care decisions [18]. Therefore, we have developed innovative methods to engage community partners in the implementation process, using the theory of planned behavior [19] to increase buy-in and motivation. Mixed methods will be used to define barriers and facilitators to implementation and create protocols guiding the ongoing use of measurement-based care in EP programs. These innovative engagement, refinement, and training processes are critical for the successful implementation of measurement-based EP care. Additionally, collaboration at the state level via EPI-CAL and the national level via EPINET lead to even greater opportunities for large-scale evaluation and research, pushing forward innovation in EP care.

### Objectives

The main aims of the study are to (1) determine the feasibility of implementing an LHCN across a diverse, decentralized network of early psychosis programs, (2) determine if the implementation of an LHCN leads to an increase in the delivery of measurement-based care, and to what extent, and (3) determine if the implementation of measurement-based care is associated with significant improvements in key outcomes.

## Methods

### Study setting and eligibility criteria

#### Study site eligibility

To participate in EPI-CAL, the program must be in California and serve individuals with EP, which can include both individuals experiencing their first episode of psychosis (FEP) and those who are at clinical high risk (CHR) for developing psychosis. All programs must have defined criteria that exclude those who do not have a recent onset of psychosis, although the specific limit on duration since the onset of their first episode can vary from program to program (see Supplementary Table S1 for specific program admission criteria).

#### Individual participant eligibility

All service users who are eligible to receive care in a participating EP program are eligible to enroll in the study. While the specific eligibility criteria for each participating program can vary (see Supplementary Table S1), all service users must have a psychosis diagnosis or be at CHR for psychosis. Those with CHR may be experiencing attenuated psychotic symptoms, brief limited or intermittent threshold psychotic symptoms that do not meet formal criteria for a psychotic disorder, or be at heightened genetic risk for a psychotic disorder paired with recent challenges in daily living [20]. It is important to note that the national EPINET initiative focuses on participants who are experiencing their first episode of psychosis, although data from individuals who are assessed to be CHR are still included. EPI-CAL includes data from CHR individuals, as most EP programs in California serve both FEP and CHR populations and data collection occurs in the context of providing regular clinical services.

Following the recruitment of the service user, support persons of the individual are also invited to participate. Support persons are broadly defined and identified by the service user; they can include a parent, grandparent, spouse/partner, sibling, or other close relationship.

### Recruitment

Recruitment is only open to service users and their support persons from participating EP programs in California that have joined the EPI-CAL network (Supplementary Table S1). Any service user who is eligible for the EP program, along with their support persons, is eligible to participate in the study and can be registered in the Beehive application by program staff. Data generated from survey completion on the Beehive application is clinically relevant, meant to be used in clinical care, and integrated into a clinic’s workflow. Therefore, EPI-CAL study staff support service user registration and enrollment in Beehive. The research team works closely with each participating EP clinic to ensure adequate integration of Beehive in clinical care in addition to demonstrating the utility of data collected through Beehive to ensure adequate recruitment of EP program participants. Use of Beehive by service users, support persons, and EP program staff does not require written informed consent, but rather a completed end-user license agreement (EULA). Trained EP program staff introduce Beehive to participants who are shown a video explaining the purpose of the study and how their data will be used. Participants are then presented with the EULA to read and make data sharing choices. Participants are not able to use Beehive until they have completed their EULA. Participants can change their data sharing permissions at any time during participation in EPI-CAL. If a service user does not opt in to sharing data for research purposes, the research team does not use data about that service user for research purposes even if the data are derived from clinic staff or support persons. The institutional review board of the University of California, Davis, reviewed and approved the study (1403828-21, see Ethics Section).

### Interventions

#### Beehive - the data collection and visualization system

**Fig. 1:**
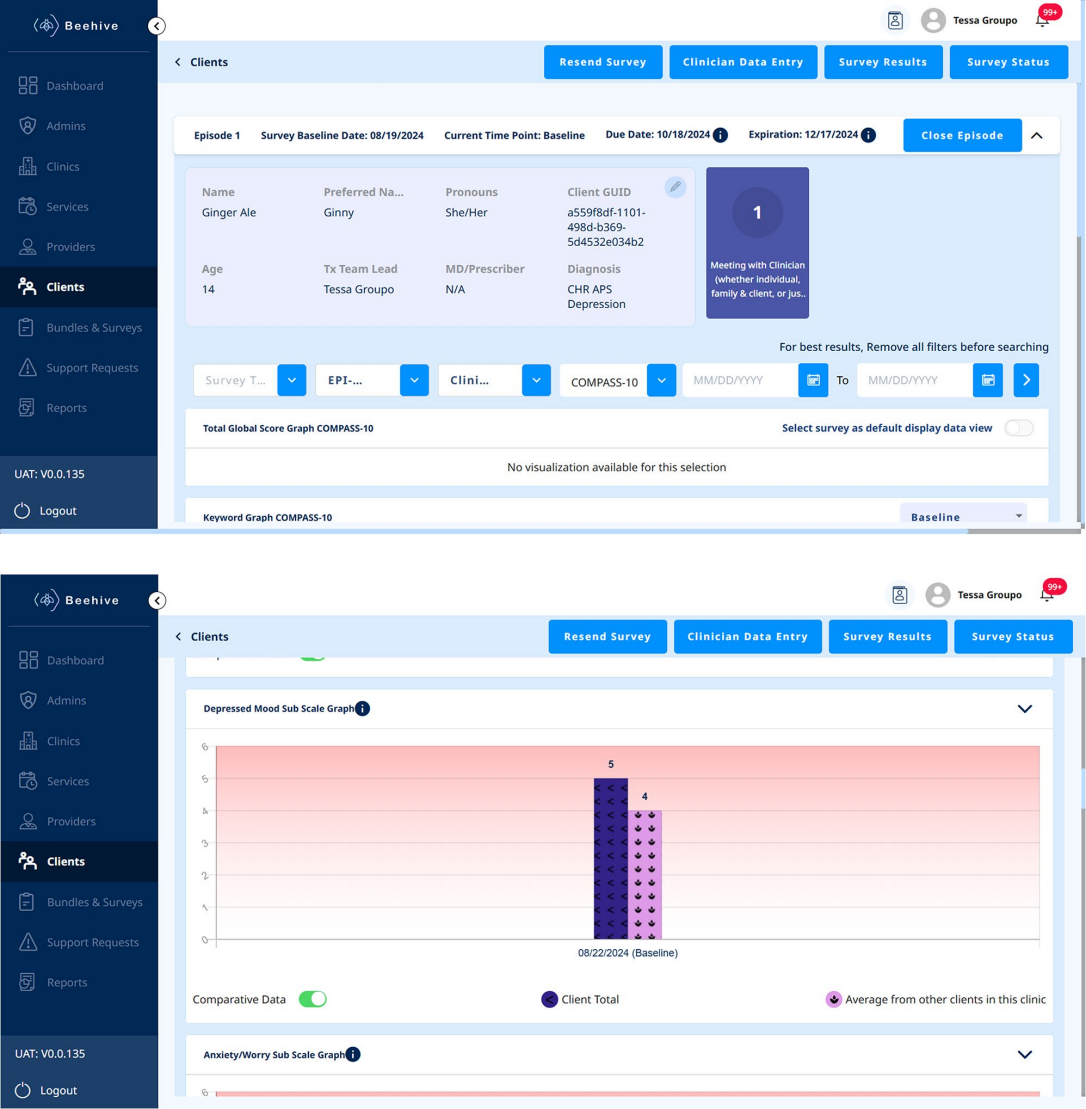
Beehive service user data page

A key component of the proposed LHCN is the integration of the data collection and visualization system we have created, called Beehive, across all clinics within the EPI-CAL network. This eHealth application was designed to: (1) collect outcomes data from service users receiving care at an EP program and their support persons (i.e., family or other close individuals who service users choose to involve in their treatment), (2) provide the data for providers on a secure web-based dashboard to support measurement-base care, and (3) allow data to be used for program or research analysis. The dashboard includes a host of features such as a comprehensive strengths-based service user assessment that can inform clinical assessment, care planning, and psychoeducation; an alert system to support providers in safety planning and assessment; and a visualization system to track outcomes over time and further inform care (Fig. 1). Additional information regarding the features of this system and the extensive co-designed development process will be described in a partnering manuscript.

#### Beehive training

**Fig. 2:**
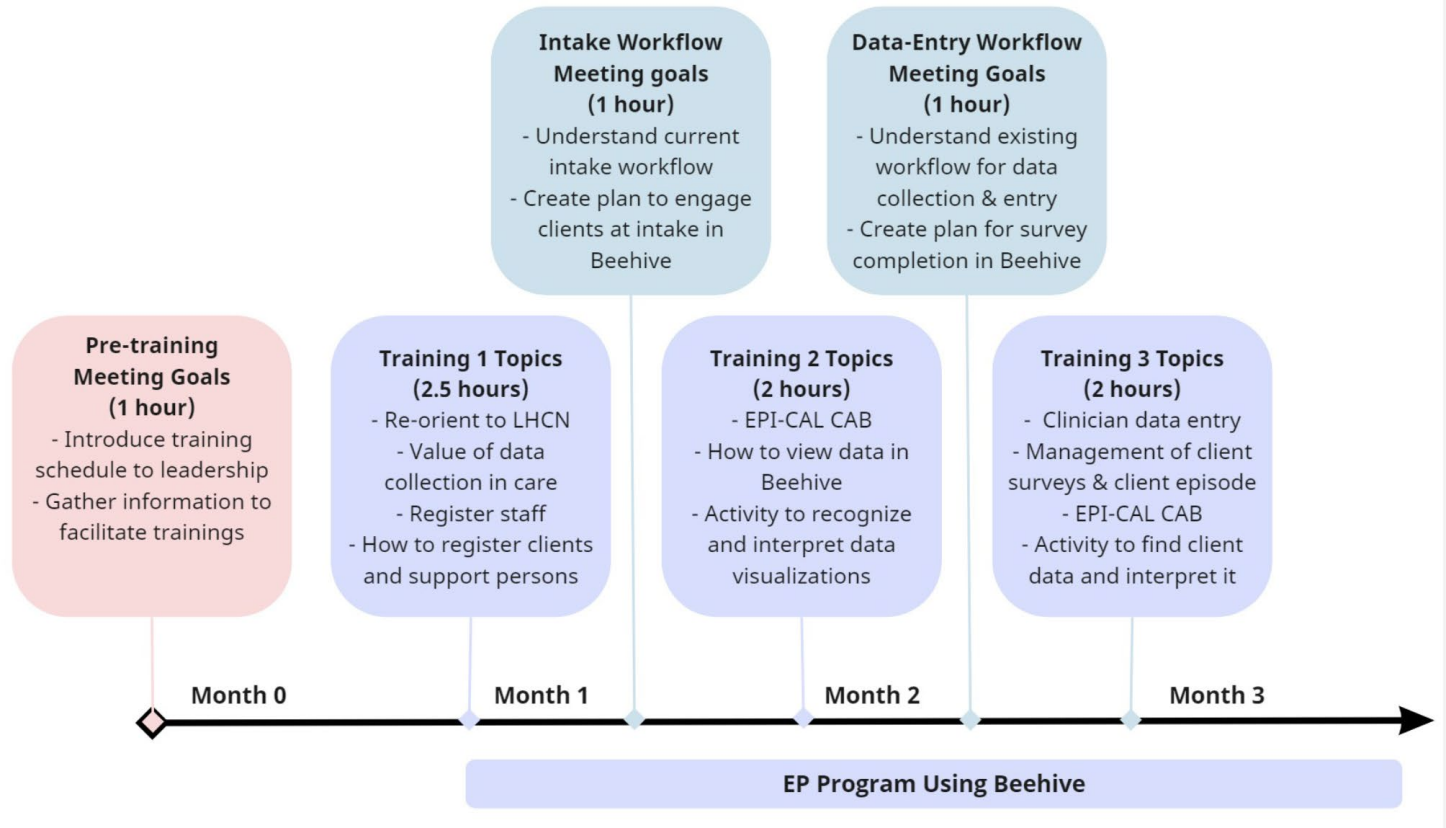
EPI-CAL EP program training schedule

All participating EP programs are trained to implement Beehive within their clinical practice. Before programs use Beehive, the research team provides training for the entire program over a period of weeks to months, depending on the availability of clinic staff. This training is most frequently offered virtually, but in-person training is also offered if requested by a program. According to the design, EP program staff members are asked to attend every training session, including program leadership, clinicians, administration, prescribers, supported education and employment specialists, peer support specialists, case managers, etc. If an individual staff member misses a specific training session, we request that the staff member review the recorded session at a later time and follow up with them to ensure that training has been completed. Training topics cover Beehive workflows, the EPI-CAL core assessment battery (CAB), and introduces how to use data in care (Fig. 2).

#### Ongoing support

Our team offers ongoing implementation support after the initial training series concludes. Each program is assigned a specific point person from the research team who works closely to support Beehive implementation. Their support may include weekly check-in meetings, on-demand problem-solving to resolve any technical barriers, training refreshers, and summaries of enrollment progress. EP program staff have access to the Beehive resource guide, a searchable wiki, and asynchronous training videos in an online learning management system for additional training and information on Beehive workflows. If programs hire new staff, our team supports their Beehive training. This may involve providing new staff with a recording of their program’s trainings, providing synchronous training directly, offering synchronous trainings open to the entire LHCN, and providing access to asynchronous training videos in a learning management system. When sites face barriers that study point persons cannot resolve, point persons bring those issues to the larger implementation team for problem solving. For example, during our initial testing phase of the application, pilot sites had questions about how to present Beehive to service users. Point persons and the wider research team created several materials, including intro scripts, infographics, and handouts to support introducing Beehive to service users as part of their regular intake process. Additional resources have also been created to specifically support the engagement of Spanish-speaking participants, including a Beehive consultation for providers working with this population and additional handouts in Spanish.

### Outcome measures and instruments

The first aim of the project is to determine the feasibility of implementing an LHCN across a diverse, decentralized group of EP programs. To determine if the LHCN has been effectively implemented (Aim 1), we record the total number of eligible service users who enroll in the EP program during the study period, the number that successfully complete the EULA [21], the number that agree for their data to be used to support research activities, and the number of service users and support persons that complete at least one survey. We expect that 70% of eligible EP program participants and 50% of their available support persons across the network will enroll and complete baseline surveys based on prior studies within an EP population [22]. To determine this, EP programs will be asked to provide our team with the total program census number annually, which is compared to service users enrolled in Beehive. Service users must have completed their EULA to be considered enrolled. Data on the number of available support persons is available in Beehive, and we can assess whether a primary support person (PSP) has completed enrollment.

We will measure survey completion of any of the surveys available in Beehive’s CAB in order to further assess implementation success. The CAB includes validated measures for both service users and their primary support person to complete. The initial proposed CAB was developed by selecting relevant measures from the PhenX toolkit [23], the Mental Health Block Grant (MHBG) minimum dataset, Mental Health Services Act (MHSA) demographic reporting requirements, and existing program evaluation measures. From there, the final measures and domains were reviewed and refined in focus groups with service users, family members, and providers conducted by our team [24], and the EPINET workgroup. The EPI-CAL CAB overlaps significantly with the EPINET CAB [25] (https://nationalepinet.org/core-assessment-battery-cab/) but differs in some domains and administration methods. See Table 1 for a comprehensive list of outcomes assessed by the CAB. In addition to the EPI-CAL CAB, service users will also be able to complete cognitive testing through Beehive annually.

We also conduct semi-structured qualitative interviews with service users and providers to assess barriers and facilitators to implementing the LHCN examining service user-, provider- and program-level barriers to enrollment and completion. Purposive sampling will be used to recruit participants across clinics where Beehive adoption and implementation has been either high or low, and with service users who have and have not received measurement-based care. Service user participants will be recruited either through clinician referral or by the research team directly by contacting individuals who had previously given permission to be contacted for future research opportunities.

To determine if implementation of the LHCN leads to an increase in the delivery of measurement-based care (Aim 2), providers will complete self-report questionnaires in the pre- and post-implementation period of the project. To examine adoption of a new technology in the EP program, we will compare providers with respect to their self-reported use of data to determine treatment choices at two timepoints: prior to Beehive implementation, and then after training in and use of Beehive. Pre- Beehive implementation surveys include Treatment Alliance and Use of Data in Care Planning in reference to specific service users. Providers will also complete the Comfort with Technology survey. The sampling frame for each site will consist of surveys about service users of currently enrolled EP providers, restricted to service users with at least six months under treatment and who had been seen in the preceding month. Beehive training materials will then be implemented consistently across participating EP programs; implementation efforts will highlight the utility of data to identify treatment goals and metrics of improvement during treatment planning and provide guidance on service user-centered ways to collaboratively review data and monitor progress during care. Then, in post-implementation, the same set of surveys are administered to EP programs who have implemented Beehive in their program for at least a year. The survey sampling strategy used in the pre-implementation period to select clinician-service user pairs will be repeated after Beehive has been implemented in the clinic for a full year, to ensure a valid pre/post comparison on this outcome. Due to expected turnover from the clinician side and discharge/exit from the program on the service user side, we will not be able to sample the same group from the pre-implementation period. However, there will most likely be some representation in the post-implementation period from respondents who participated in the first phase of surveys. Therefore, these are not completely independent samples, nor are they completely repeated samples.

To determine the extent to which providers utilize the Beehive platform to deliver measurement-based care (Aim 2), we adopt two different approaches. First, we will examine whether a service user’s treatment team lead reviews completed surveys in Beehive, which is recorded by the application. During service user registration in Beehive, EP providers designate the service user’s treatment team lead, typically their primary clinician. Once a survey respondent completes a survey in Beehive, the data are immediately available to view in the Beehive dashboard. The Beehive survey reports will include a variable that shows whether or not each survey has been viewed by the service user’s treatment team lead (binary yes/no). Research staff will use this data to determine the degree to which providers are actively viewing data collected in Beehive.

To explore how Beehive data are used by those who frequently utilize the application, two types of in-app queries were developed: urgent clinical issues and data-use questions. Urgent clinical issues are a type of notification (in-app and email) in Beehive that encourages EP staff to review service user data. These notifications trigger if, during registration or survey completion, a service user endorses risk-to-self or risk-to-others on the Modified Colorado Symptom Index (MCSI) [26], the intent to stop taking their medication [27], or lack of a permanent address. Beehive displays urgent clinical issues on a dashboard widget and to resolve them, users must indicate how they used the data in care. Additionally, an in-app query is presented to the service user’s treatment team lead every ten visits to the service user’s data page. Data-use questions assess (1) if the data was reviewed during a session with the service user or support persons and, if yes, (2) how the data was used as part of care.

We will then examine whether providers’ implementation of measurement-based care is associated with significant improvements in key outcomes (Aim 3). To do this, we will compare adjusted between-groups mean differences in baseline to 12-month change in surveys available in the Beehive CAB, including the MCSI. Groups will be defined by clinician metrics from Beehive described above and assessed during this 12-month period. The MCSI is a 14-item, self-report scale designed to assess the frequency of psychiatric symptoms related to psychosis, mood, desire to hurt oneself and others, cognition and forgetfulness. Each item is scored on a 0–4 Likert-style scale and added together to give a score between 0 and 56, with higher scores indicating greater emotional distress. Reduction in score over time indicates clinical improvement.

**Table 1:** EPI-CAL outcomes collected in beehive

Domain	Respondent	Measure and/or Source*	Timepoint
Demographics & Background	Service user	- EPI-CAL team	Enrollment
Demographics and Background	Service user	- EPI-CAL researchers - California State Required Demographics Reporting - Modification from EPINET version of this question “Are you a veteran?” required question for PEI/INN Reporting - A question measures a risk factor for persistent poverty [28]. - An item was created by the EPI-CAL team and assesses factors which put a person at increased risk for homelessness [29] - Part of EPINET CAB [25] (https://nationalepinet.org/core-assessment-battery-cab/)	Enrollment
Primary Caregiver background	Service user	- EPI-CAL researchers and EPINET CAB [25]	Enrollment
Traumatic Events and Experiences	Service user	- Pediatric Adverse Childhood Experiences (ACEs) and related life events screener (PEARLS) [30]	Enrollment
Demographics and Background	Service user	- A question measures a risk factor for persistent poverty [28]. - An item was created by the EPI-CAL team and assesses factors which put a person at increased risk for homelessness, as described in literature [30] - Part of EPINET CAB [25]	Every 6 months (including Baseline)
Education	Service user	- Homelessness Risk item created by EPI-CAL team derived from literature review [29]	Every 6 months (including Baseline)
Employment and Related Activities	Service user	- EPI-CAL researchers and EPINET CAB [25]	Every 6 months (including Baseline)
Social Relationships	Service user	- Attachment Item from Social Provisions Scale [31] - EPI-CAL researchers - Distress Disclosure Index [32]	Every 6 months (including Baseline)
Family Functioning	Service user and PSP	- SCORE-15 Index of Family Functioning and Change [33]	Every 6 months (including Baseline)
Legal Involvement and Related	Service user	- EPI-CAL researchers and EPINET CAB [25]. Response options were informed from literature (35) and community partner feedback during focus groups [24].	Every 6 months (including Baseline)
Substance Use	Service user	- EPINET CAB	Every 6 months (including Baseline)
Medication, Side Effects, and Treatment Adherence	Service user	- Adherence Estimator - Glasgow Antipsychotic Side-effect Scale (GASS) [35] - Brief Adherence Rating Scale (BARS) [36] - Additional items derived from focus group feedback and written by EPI-CAL team	Every 6 months (including Baseline)
Intent to Attend and Complete Treatment Scale	Service user	- Intent to Attend and Complete Treatment Scale [27]	Every 6 months (including Baseline)
Symptoms	Service user	- Modified Colorado Symptom Index (MCSI) [26]	Every 6 months (including Baseline)
Recovery	Service user	- Questionnaire about the Process of Recovery (QPR) [37]	Every 6 months (including Baseline)
Life Outlook	Service user	- A question was derived from suggested questions from Nev Jones (personal communication, August 2020) to capture role satisfaction - Question 1 from Personal Wellbeing Index [38] - Construct prioritized in outcomes focus groups	Every 6 months (including Baseline)
Hospitalizations	Service user	- EPINET CAB	Every 6 months (including Baseline)
Traumatic Events and Experiences	Service user	-Life Events Checklist (LEC-5) [39] and PTSD Checklist for DSM-5 (PCL-5) [40]	Every 6 months (including Baseline)
Traumatic Events and Experiences	Service user	- Child and Adolescent Trauma Screen (CATS) – Youth Report (Age 7–17) [41]	Every 6 months (including Baseline)
Shared Decision Making and Treatment Satisfaction	Service user	-Shared Decision Making Questionnaire (SDM-Q-9) [42] - Kickstart Satisfaction: domain required for primary aims	Every 6 months (including Baseline)
Pathways to Care	Clinician	- EPINET CAB [25]	Enrollment
Diagnoses and Duration of Untreated Psychosis (DUP)	Clinician	- EPI-CAL modified this survey from EPINET CAB [25] to include more specific and exhaustive list of DSM-5 diagnoses	Every 6 months (including Baseline)
Family and/or Support Person Involvement	Clinician	- EPINET CAB [25]	Every 6 months (including Baseline)
Risk to Self/Others	Clinician	- EPI-CAL researchers modified from EPINET CAB [25]	Every 6 months (including Baseline)
Health	Clinician	- EPI-CAL researchers modified from EPINET CAB [25]	Every 6 months (including Baseline)
Medications	Clinician	- EPINET CAB [25]	Every 6 months (including Baseline)
Service Use	Clinician	- EPI-CAL researchers	Every 6 months (including Baseline)
Functioning	Clinician	- Either Global Functioning: Role [43] and Global Functioning: Social [44] or MIRECC GAF [45]	Every 6 months (including Baseline)
Symptoms	Clinician	One of: - Brief Psychiatric Rating Scale (BPRS) [46] - Positive and Negative Symptoms of Schizophrenia Scale (PANSS-6) [47] - COMPASS-10 [48]	Every 6 months (including Baseline)
Demographics and Background of Primary Support Person (PSP)	PSP	- A question included to measure exposure to poverty at a young age, which was indicated as a risk factor for persistent poverty [28]. - A question derived from ABCD Study [49] (https://abcdstudy.org) and Deanna Barch (Personal Communication, September 2020) - Collateral report for the service user-self report question. Response options were informed from literature [34] and stakeholder feedback during focus groups.	Enrollment
Demographics and Background of Primary Support Person	PSP	- EPI-CAL Researchers	Every 6 months (including Baseline)
Legal Interactions and Related	PSP	- Collateral report for the service user-self report question. Response options were informed from literature [34] and stakeholder feedback during focus groups.	Every 6 months (including Baseline)
Family Impact	PSP	- Burden Assessment Scale [50]	Every 6 months (including Baseline)
Symptoms	PSP	- Modified by EPI-CAL team for collateral report from original MCSI [26]	Every 6 months (including Baseline)
Medications	PSP	- Modified by EPI-CAL team for collateral report from original GASS [35]	Every 6 months (including Baseline)

^*For measures without a single validated source, our team and other collaborators created the questions based on multiple sources^

#### Program fidelity

In addition to the program-level data described here, we also collect project data via fidelity assessments, program surveys, and the program level core assessment battery (PL-CAB). Each program completes a fidelity assessment to determine the components of coordinated specialty care (CSC) provided using the First Episode Psychosis Services Fidelity Scale (FEPS-FS) [51], a standardized measure of fidelity to EP program best practices. Similar to the fidelity assessments, program surveys and the PL-CAB assess various components offered through the CSC program, program census, and staffing. The data from these other sources may also be used to inform the analysis of the program-level data.

### Data collection timeline

Participants, support persons, and EP program staff complete CAB clinical outcomes surveys at treatment baseline and every six months throughout treatment in the EP program. EP program staff also enter data at the time of the service user’s discharge from the EP program. Survey windows and due dates are all tied to the survey baseline date that program staff enter for each service user. For our purposes, the survey baseline date is associated with a service user’s start of care in their program, not when they are enrolled in Beehive. Though different programs may use a different visit during the early phase of engagement in clinical care as the anchor for the survey baseline date, all the service users within any given clinic use the same type of clinical visit as the baseline benchmark.

**Fig. 3:**
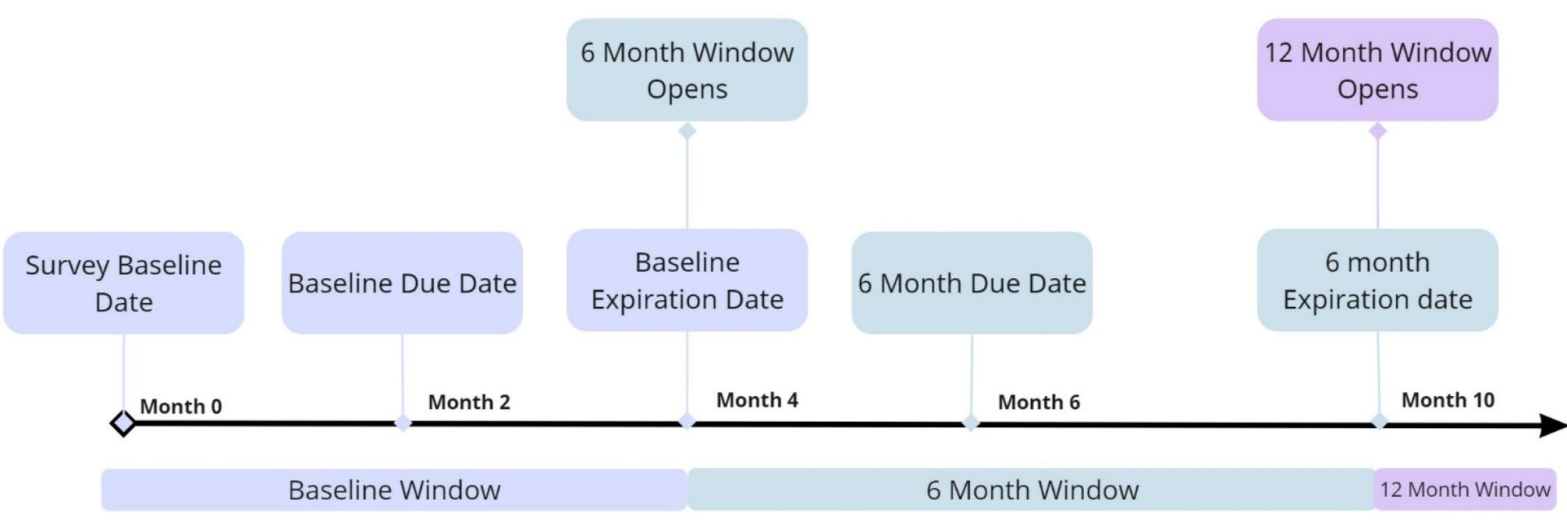
Beehive survey windows

Beehive survey windows span several months each to facilitate the completion of the CAB. The baseline window in Beehive starts at the survey baseline date and ends 4 months later. Subsequent follow-up windows are due every six months from the survey baseline date. The follow-up windows open two months before the due date and close 4 months after the due date (Fig. 3). Programs are encouraged to complete surveys as close to the due date as possible, but flexibility is given to accommodate delays in clinical visits or unexpected barriers to completion of the survey (i.e., service user crisis or hospitalization). Data collection continues every six months throughout a service user’s treatment.

### Sample size

Our estimated sample size is based on each EP program’s estimate of new clinical intakes in their program per year. We estimate that we will be able to systematically collect outcomes data on approximately 500 FEP service users and 300 additional CHR service users over the course of the study. Our target sample size for the barriers and facilitator interviews is 30 providers and 30 service users, or at the point where the main themes meet saturation. There are also additional participants in EPI-CAL from various focus groups, qualitative interviews, surveys outside of Beehive, and fidelity assessments.

### Data collection methods

EPI-CAL researchers consulted the Guidelines for Data Acquisition, Quality and Curation for Observational Research Designs (DAQCORD) [52] on the design and implementation of this protocol to achieve a high-quality data set. Whenever possible, DAQCORD indicators were implemented through Beehive. We will highlight our implementation of several DAQCORD indicators below. See Supplementary Table S2 for the full list of DAQCORD indicators and how they have been implemented in this study.

Regarding the DAQCORD indicator of internationalization, Beehive and the EPI-CAL CAB will be localized into 12 threshold languages to meet the requirements of operating in California Medicaid (Medi-Cal) clinics. The languages will include English, Spanish, Vietnamese, Mandarin, Cantonese, Farsi, Armenian, Hmong, Khmer (Cambodia), Korean, Russian, and Tagalog. All measure translations go through either back-translation or translation by committee.

The majority of survey data collected for EPI-CAL is collected directly from service users and their support persons through Beehive and is described in the outcomes section. We designed Beehive to facilitate service users and support persons completing these surveys independently, though we encourage clinical staff to support individuals as needed (e.g., answering questions about survey content). There are some outcomes that require clinical staff to input data into Beehive (e.g., diagnosis, service use) and several outcomes of interest (e.g., symptoms, functioning, suicide risk) are collected from multiple respondent types.

Regarding the DAQCORD indicator to test data collectors and provide them with feedback regarding the accuracy of their performance across all relevant study domains, EPI-CAL clinical psychologists oversee the training of program staff who will complete the COMPASS-10 [48] and Global Functioning: Social [44] and Role [43] scales. Individuals either attend the live training or can watch a recording of it. Program staff must then provide a rating for 10 vignettes and must score within 1 point of the gold standard rating for each vignette to pass. We provide feedback for incorrect answers and allow program staff to retake the test with a different set of vignettes if needed.

### Data management

For data collected outside of Beehive, including program census information collected to assess the feasibility of implementing an LHCN, data are collected from programs and entered into an Excel document and stored on a HIPAA-compliant cloud storage.

Regarding the EPI-CAL CAB, since participants enter survey data directly into Beehive, data entry by research staff is not required. Most data cannot be edited or modified after it is entered. For example, after the first survey has been completed about a service user, the survey baseline date can no longer be modified by the user. Any requests to change this field must be approved and implemented by the research team so that they can ensure all data are automatically assigned to the appropriate time point by the application. Additionally, surveys are in a read-only state after they have been completed by any type of survey respondent (service user, primary support person, and clinical team).

The database is hosted on Amazon Web Services (AWS) cloud. Amazon’s Simple Storage Service (Amazon S3) is used for storing content, logs, and backup data. This allows versioning, secured storage, and retrieval. Server-side data encryption is managed using AWS S3. Report functionality in Beehive allows data to be saved as a .csv file. The research team can access data across all programs but have access to only a limited data set. The only protected health information (PHI) that the research team has access to is a service user’s zip code, month and year of birth, and dates of service. When data is exported from Beehive by the research team, it is stored according to UC Davis policies for storage of PHI.

Because the data collection is happening in the context of regular clinical service, EP programs also have access to the data from their own program. Access to data is restricted based on user permissions managed by the EP programs. When programs download data from Beehive, it is their responsibility to treat the data as sensitive data and protect that data according to federal and state regulations and institutional requirements.

Information about all variables is documented in a data dictionary. The data dictionary documents: (a) variable name, (b) date of implementation for each translation, (c) a description of the data stored under the variable name, (d) the type and format of data (e.g., numeric, date MM/DD/YYYY), (e) implemented data validation, (f) response options when relevant, (g) missing data code, (h) validly skipped data code, (i) scoring information, and (j) details about skip logic when relevant. The data dictionary is updated whenever changes are made to the database, for example when new variables or new translations of existing variables are added.

Data validation is built into the data collection process in Beehive. Whenever possible, data collection has been designed to avoid free text fields so that data validation can be implemented. Some examples of data validation implemented in Beehive data collection are (a) minimum and maximum values for numeric text entry data, (b) formats for dates and zip codes, etc., (c) restriction on certain response options that are part of select-multiple questions (e.g., cannot select “none of the above” and select any other option).

### Data analysis

Prior to analysis, we will complete descriptive summaries for all data collected in Beehive, including service user and clinician demographics, survey completion for each survey at each timepoint, and survey scores for quantitative measures. The distribution and completeness of each analysis variable will be examined to determine appropriateness of different statistical methods. The availability of within-person longitudinal data will be reviewed to determine whether longitudinal or cross-sectional approaches are most appropriate. Descriptive summaries will be generated for each clinic individually, as well as network wide.

To address Aim 1, we will examine whether we achieved adequate enrollment in Beehive by using descriptive statistics to see if at least 70% of eligible participants and 50% of their available support persons across the network were enrolled and completed at least one survey timepoint. To approximate the number of total service users eligible for enrollment, we will pull the total census number from each program annually. Eligible service user participants are defined as those who are determined eligible to receive care at each program. Available support persons are defined and identified by the service user. Service users must have completed their EULA to be considered enrolled. For the analysis, we only consider individuals who have agreed to share data with the University of California, Davis (UCD) as “enrolled”, but service users can decline this option and still use their data within their program for clinical purposes. Just like service users, primary support persons are not considered enrolled unless they have agreed to share data with UCD. Service users and support persons can make different choices regarding their data sharing permissions, i.e., a service user can decline to share their data for research purposes while a support person can opt-in. For the feasibility analysis, we will only examine what proportion of enrolled service users also have an enrolled PSP, acknowledging that there may be more enrolled PSPs whose corresponding service user opted out of data sharing. Survey data analysis procedures for clustered data (treating EP programs as clusters) will summarize characteristics of enrolled service users who complete enrollment and at least one survey. Enrollment rates (with 95% confidence interval) will be computed for (1) all eligible service users and (2) potentially available support persons. For the latter, we will report, for the denominator of eligible service users with available support persons, what proportion of those service users had at least one support person complete a baseline or 6-month survey assessment.

To assess Aim 2, the adoption of Measurement-Based Care (MBC), we will first examine the degree of use of data in care between the pre- and post-implementation periods of the project. Before Beehive implementation in each EP program, providers complete pre-implementation surveys about their demographic information (age, sex, race, ethnicity) and professional characteristics (years of education, degree type) and complete questionnaires on their (1) beliefs about the utility of data in care planning and (2) skills in discussing data with service users. Compared to the pre-implementation period, we hypothesize that providers will report a change in the use of data to determine treatment choices after training and using the app for at least one year. Separate models will be fit for each of the primary and alternative operationalization of Beehive clinician-usage metrics as the exposure variable of interest. Adoption of data in care will also be measured by examination of whether a service user’s treatment team lead examined completed surveys from service users. To determine the degree to which providers are actively viewing data collected in Beehive, research staff will review the Beehive survey reports variable that shows whether or not each survey has been viewed by the service user’s treatment team lead (binary yes/no). We hypothesize that EPI-CAL treatment team leads will have viewed service user data collected through Beehive in at least 50% of cases. Lastly, we will examine whether the clinician reported that Beehive data impacted the treatment plan as assessed by the in-app queries periodically presented to EP provider users.

Through the qualitative work that was completed in the first phase of this project [24], a variety of key outcomes were identified by our program, service users, and support person workgroups. Psychiatric symptoms, quality of life, and functioning were prioritized as key outcomes by all types of respondents and our analysis will center on these domains. When examining group-level differences, it is important to note that there is not a “Beehive” and “not Beehive” group of service users; all service users are assigned to the Beehive group and thus no analysis can examine the effect of Beehive use in treatment compared to a typical control group. Instead, to assess the impact of utilizing MBC in an EP LHCN (Aim 3), we will analyze a dataset consisting of one record per service user per follow-up assessment timepoint and outcomes expressed as within-person change scores from baseline (for continuous measures) or as count or binary outcomes. For count or binary outcome data, the corresponding baseline value of the outcome will be included as a person-level covariate, when appropriate. Outcomes will be measured by the MCSI, personal wellbeing index (PWI), and functioning measures (Global Functioning Social and Role Scales (GF: S and GF: R) or Mental Illness Research, Education, and Clinical Center (MIRECC) version of the Global Assessment of Functioning (GAF) scale) for each of the six-monthly assessment timepoints during the first 24 months. Continuous outcomes will be transformed into within-person change scores from the baseline assessment for each follow-up assessment. Data are structured hierarchically; there is nesting of measurements from service users, who are nested within clinicians within EP programs. Therefore, for continuous, binary, and count outcomes, generalized linear mixed models will be used to estimate the adjusted effects of exposures on the key outcomes of interest, adjusting for a parsimonious set of other clinician- and service user-level covariates. Random effects will be specified for sites, with additional effects specified for clinician and service users’ contribution to the model fit, according to the Schwarz Information Criterion.

A key operationalization of the exposure indicator will be based on a composite indicator for any review of the service users’ completed surveys. In particular, this variable will be scored a 1 for a given service user in a given follow-up assessment if the treatment team lead reviewed the service users’ completed survey data. The comparison groups are defined by clinician metrics from Beehive aggregated over the 6-, 12- and 18-month assessment period, and the primary analysis is based on a composite indicator for any review of the service users’ survey data by the treatment team lead. We will also assess timepoint-specific changes in psychotic symptom severity for each of the half-yearly assessment timepoints during the first 24 months, with the primary analysis based on a time-varying indicator for any endorsement of “impact on treatment plan” on the in-app queries as a time-varying independent variable.

#### Analysis of population and missing data

All EP program service users are eligible to participate in the study and complete surveys on Beehive. To address attrition in longitudinal survey completion and missing data, we will use multiple imputation to impute follow-up assessment scores and change scores based on them, as appropriate.

#### Qualitative analyses

Though qualitative data is not be directly linked to Beehive user IDs, we also use interview data to examine service user-, provider- and program-level barriers and facilitators to enrollment and completion via semi-structured qualitative interviews with service users and providers. Service user-, provider- and program-level implementation barriers will be identified utilizing an inductive approach to thematic analysis. Purposive sampling will be used to recruit participants of service users and providers across clinics where Beehive adoption and implementation has been both high and low, and with service users who have and have not received measurement-based care. Multiple coding will be adopted, and where possible, service users and providers will be involved in developing the topic guide and reviewing the data analysis and interpretation.

### Data monitoring

Research staff monitor the enrollment of eligible participants on a weekly basis and discuss progress with EP programs at regular meetings to facilitate enrollment of all potential participants. Our internal data management team monitor data as it is collected by sites in Beehive, such as service user enrollment and completion of the MCSI, to ensure data quality. We also periodically monitor datasets exported from Beehive for statistical analyses, including evaluating individual survey data, for missing or incomplete data. We provide feedback to sites, such as following up about missing diagnoses, if applicable. We conduct an ongoing analysis of the enrollment of service users and support persons solely to ensure that the study is appropriately reaching the target population.

#### Formal committee

In addition to our study team that performs regular monitoring of study data, we have also established an Executive Committee meeting that meets annually to review study progress and interim analysis results. The Executive Committee consists of the principal investigator, co-investigators, and key consultants.

#### Interim analysis

Formal interim analyses are not needed to assess the trial intervention on an experimental group as all eligible service users are eligible to participate in the study.

## Ethics and dissemination

### Research ethics approval

The institutional review board of the University of California, Davis, approved the study (1403828-21, California Collaborative Network to Promote Data-Driven Care and Improve Outcomes in Early Psychosis [CORE]). Additionally, several of the counties and universities with a program participating in EPI-CAL required a separate review and approval of the project by their institutional review board or compliance department.

Some EP programs require approval by additional oversight bodies associated with California counties, universities, and/or community-based organizations. These oversight bodies review and approve research prior to initiation of any research activity the EP programs they oversee.

### Protocol amendments

Any modifications to the protocol that materially impact the study are reviewed and approved by UC Davis IRB before implementation. If there are significant changes to the study plan, such an amendment will be agreed upon by the project’s sponsors including the NIMH, the MHSOAC, the California county sponsors, and One Mind. All changes to the protocol, forms, and study documentation are first approved by UCD IRB before being presented to the other oversight bodies that are involved in this project.

### Consent or assent

Written consent is obtained from study participants for the following study-related components: focus groups, feedback interviews, fidelity assessments, and surveys completed by service users, support persons, and EP program staff. Assent is obtained for minors, with their legal guardian signing the consent on their behalf. Asynchronous written consent in English and Spanish will be obtained for cognitive testing. If a participant does not speak English and we have the language capacity on our internal team to conduct the informed consent process, the consent and/or assent forms is translated into the participants’ preferred language by UC Davis Medical Interpretive Services and the consent is conducted in that language. We follow standard informed consent practices and EPI-CAL research staff will be responsible for administering the consent and ensuring all documentation is completed. Every research staff member is required to complete all required HIPAA and CITI training, certificates of which are kept on file for all personnel, prior to engaging in human subjects research activities.

### Ancillary studies

Data collected for this research may also be used for future research studies, which is described in the EULA that participants complete when they make decisions about sharing their data for research. We do not share any personally identifiable information. Our goal is to make more research possible. These studies may be done by researchers at this institution or other institutions. Data may be placed in one or more external scientific databases for access and use. We will not ask participants for additional permission to share de-identified information. Efforts are be made to limit the use or disclosure of participant personal information to people who need to review this information.

### Confidentiality

Data is primarily stored at UC Davis; some data are also be stored at UCSF and UCSD with similar protections outlined below. All information collected does not include any names or references to individual service users, support persons, or staff or personal health information. The only PHI that the research team has access to from Beehive data is the service user’s zip code, month and year of birth, and dates of service. No health records or identifiable information should be shared with our team, except to facilitate staff interviews. The study investigators and primary research team are the only ones who will have access to the data. We regularly release data to the ENDCC with the following stipulations: (a) dataset only includes service users who have given their permission for us to share it with ENDCC, (b) dataset does not include any identifiers or free-text fields, (c) dataset does not include the study ID assigned to the service user in Beehive. For the electronic files and datasets, copies of each file will be maintained on HIPAA-compliant cloud storage, e.g., UC Davis’s OneDrive. All copies of these electronic files are also encrypted. Data will be stored for after the end of the project to allow ongoing data analysis and publication. All oversight of data sharing will be provided by the project manager and the Principal Investigator Dr. Niendam. E-mailing of files is only allowed if data is de-identified and can be sent via encrypted, password-protected messaging.

### Dissemination

#### Dissemination plan

Our team plans to disseminate the results of this project in a variety of ways. Results and information from the clinical trial are submitted to ClinicalTrials.gov as outlined in the policy and according to the specific timelines stated in the policy (identifier NCT04007510; registered 07/05/2019). Results of the study will also be published in peer-reviewed academic journals or presented at conferences to share our findings with the larger community. Products from this project (e.g., webinars, written products, presentations) are available on the EPI-CAL website (https://epical.ucdavis.edu/). Results of the evaluation will also be communicated with our EP program partners via biannual advisory committee meetings, deliverables, annual reports, or larger presentations based on the needs of our partners.

#### Publication policy

We established an EPI-CAL Publications Committee and corresponding publication guidelines to ensure that (1) the rich data derived from EPI-CAL are used to their full productivity, (2) publications are not iterative, and each publication makes the best use of the full data set, (3) project resources are used wisely, (4) researchers’ intellectual ideas are respected. Authorship for publications derived from EPI-CAL data is determined following the International Committee of Medical Journal Editors guidelines. There is no intention for the use of professional writers on authorship of work derived from project data.

## Discussion

The goal of the current study is to assess the feasibility of establishing a Learning Health Care Network of EP clinics in California that implement harmonized data collection using a custom-built application, Beehive. EPI-CAL’s LHCN aims to improve coordination and increase knowledge sharing across heterogenous EP programs, including university- and community-based CSC programs. Prior to implementation of the EPI-CAL LHCN, there has not been a unified attempt to harmonize core data metrics and outcome measures across the disparate EP programs in California. The core battery of evidence-based measures utilized in EPI-CAL’s LHCN focuses on treatment outcomes of importance to service users, providers, and support persons as the survey battery was designed with extensive input from those community partners.

The harmonized core data metrics collected across California EP programs are collected using a unique data collection and visualization application, Beehive. EP program providers will need sufficient training and support to implement a new method of data collection into their clinical practice, especially given the already high workloads and reporting requirements EP programs face. Our approach to a successful implementation of measurement-based EP care includes detailed training, ongoing access to training and resource materials, and consistent support via EPI-CAL team members. We conduct barriers and facilitator interviews to evaluate the effectiveness of the implementation process and refine our approach accordingly.

While collecting a harmonized dataset of key outcome measures is a major goal of EPI-CAL LHCN, the heterogeneity across programs enables detailed analysis of specific program elements that lead to program success and improved treatment outcomes for service users within the network. Specific program elements will be evaluated by conducting a fidelity assessment with each program. In addition to program elements, we will also examine if treatment outcomes vary based on clinician’s use of data in care. For example, we will examine treatment outcome differences across service users whose treatment team lead examines the service user-level data available in Beehive and whether they incorporate this data into care decisions. We will include relevant clinician metrics in our analyses of treatment outcomes, which include clinician demographic information collected at registration, such as degree level, and years working with this specific population.

Beyond establishing the California Learning Health Care Network of EP programs, EPI-CAL will provide a harmonized dataset to the EPINET initiative for the purpose of advancing early psychosis care, improving recovery outcomes, and scientific discovery. The contribution of outcomes data from the California network to the national level via EPINET will improve the development of EP care, early intervention, and provide even greater opportunities for large-scale evaluation and research of EP care.

## Electronic supplementary material

Below is the link to the electronic supplementary material.

Supplementary Material 1

Supplementary Material 2

## Data Availability

No datasets were generated or analysed during the current study.

## Abbreviations

ABCD: Adolescent brain cognitive development
AWS: Amazon web services
BARS: Brief adherence rating scale
BPRS: Brief psychiatric rating scale
CAB: Core assessment battery
CATS: Child and adolescent trauma screen
CHR: Clinical high risk
CITI: Collaborative institutional training initiative
COMPASS-10: Computerized prescriber support system- 10 item
CSC: Coordinated specialty care
DAQCORD: Data acquisition, quality and curation for observational research designs
DSM-5: Diagnostic and statistical manual of mental disorders, fifth edition
ENDCC: EPINET national data coordinating center
EP: Early psychosis
EPI-CAL: Early psychosis intervention network of California
EPINET: Early psychosis intervention network
EULA: End user license agreement
FEP: First episode of psychosis
FEPS-FS: First episode psychosis services fidelity scale
GASS: Glasgow antipsychotic side-effect scale
GF: R: Global functioning: Role
GF: S: Global functioning: Social
HIPAA: Health insurance portability and accountability act
INN: Innovations
IRB: Institutional review board
LEC: Life events checklist
LHCN: Learning health care network
MBC: Measurement-based care
MCSI: Modified colorado symptom index
MHBG: Mental health block grant
MHSA: Mental health services act
MHSOAC: Mental health services oversight and accountability commission
MIRECC GAF: Mental illness research, education, and clinical center global assessment of functioning
NIMH: National institute of mental health
PANSS-6: Positive and negative symptoms of schizophrenia scale − 6 item
PCL-5: PTSD checklist for DSM-5
PEI: Prevention and early intervention
PHI: Protected health information
PL-CAB: Program level core assessment battery
PSP: Primary support person
PTSD: Post traumatic stress disorder
PWI: Personal wellbeing index
QPR: Questionnaire about the process of recovery
SCORE-15: Systemic clinical outcome and routine evaluation − 15 item
SDM-Q-9: Shared decision making questionnaire − 9 item
UCD: University of California, Davis
UCSD: University of California, San Diego
UCSF: University of California, San Francisco
