# Supplementary material for: The California collaborative network to promote data driven care and improve outcomes in early psychosis (EPI-CAL) project: rationale, background, design and methodology
Source: BMC Psychiatry. 2024 Nov 14;24:800. doi: 10.1186/s12888-024-06245-6 (PMC11566177; doi:10.1186/s12888-024-06245-6)
Supplement: Supplementary file 1 — Supplementary Material 1 [file 12888_2024_6245_MOESM1_ESM.docx]

# Supplementary Table 2: DAQCORD Indicators as Implemented in EPI-CAL and Beehive

| **Study Phase** | | **Indicator** | **EPI-CAL/Beehive implementation** |
| --- | --- | --- | --- |
| Design-time | 1 | The case report form (CRF) has been designed by a team with a range of expertise | *CRF was designed collaboration with the entire research team. Experts in specific content areas were contacted for their input on designing certain questions (i.e., measuring experience of poverty, role satisfaction, social satisfaction, risk factors for homelessness).* |
| Design-time | 2 | There is a robust process for choosing and designing the dataset to be collected that involves appropriate stakeholders, including a data-curation team with appropriate skill mix | *Process for choosing and designing the data set involved multiple meetings with entire EPI-CAL research team, input from EPINET steering committee, and input from 20+ focus groups with community partners.* |
| Design-time | 3 | The data ontology is consistent with published standards (common data elements) to the greatest extent possible | *We employed validated measures whenever available. Next, we used all of the EPINET-written measures for consistency with other EP hubs. Finally, we created questions when no appropriate validated measures available for outcomes not assessed in EPINET battery.* |
| Design-time | 4 | Datatypes are specified for each variable | *Data types are specified in the data dictionary.* |
| Design-time | 5 | Variables are named and encoded in a way that is easy to understand | *We used a team approach to determine variable names. We have made adjustments as necessary to enhance clarity (e.g., when we have added additional versions of variables to the data dictionary).* |
| Design-time | 6 | Relational databases have been appropriately normalized: steps have been taken to eliminate redundant data and remove potentially inconsistent or overly complex data dependencies | *The team reviewed core assessment battery for redundancies and removed whenever there were non-distinct data.* |
| Design-time | 7 | Each individual has a unique identifier | *Globally Unique Identifiers (GUIDs) are used for each user in the application. Data which is submitted to ENDCC for NIH data archives are assigned an NIH pseudo GUID (pGUID).* |
| Design-time | 8 | There is no duplication in the data set: data has not been entered twice for the same participant | *Beehive prevents this. For example, the same user cannot be added again to the same clinic. For example, service users may only complete a survey once at each timepoint.* |
| Design-time | 9 | Data that is mandatory for the study is enforced by rules at data entry and user reasons for overriding the error checks (queries) are documented in the database | *Data is locked after submission. All text entry fields include data validation when relevant (for example, date vs. zip code vs. any characters).* |
| Design-time | 10 | Missingness is defined and is distinguished from "not available," "not applicable," "not collected," or "unknown" | *We have included response options of "prefer not to respond," "unsure/don't know" and "not applicable" when appropriate. In the data reports, there are codes to distinguish “missing” data from “validly skipped” data.* |
| Design-time | 11 | Range and logic checks are in place for CRF response fields that require free entry of numeric values. Permissible values and units of measurement are specified at data entry. | *In the situations where text entry is the most appropriate response option, we have enforced data validation such as date format, numeric validation, and zip code validation.* |
| Design-time | 12 | Free text avoided unless clear scientific justification and (e.g.) qualitative analysis plan specified and feasible. | *Whenever possible, we designed the core assessment battery to avoid text entry responses.* |
| Design-time | 13 | Database rule checks are in place to identify conflicts in data entries for related or dependent data collected in different CRFs or sources | *Beehive enforces rules to ensure that incompatible choices are excluded between related elements (i.e., setting up age-specific bundles to avoid non-applicable questions)* |
| Design-time | 14 | There are mechanisms in place to enforce/ensure that time-sensitive data is entered within allotted time windows | *Partially completed surveys must be restarted after 12 hours. Survey windows, while large, are clearly defined as 2 months prior to the due date (with the exception of baseline window, for which data cannot be completed before the baseline date) and 4 months after the due date. Database flags when CRF completion is due and overdue.* |
| Design-time | 15 | There is clear documentation of interdependence of CRF fields, including data entry skip logic | *Documented in word doc version of CRF as well as within Beehive and in the data dictionary.* |
| Design-time | 16 | Data collection includes fields for documenting that participants meet inclusion/exclusion criteria | *Documentation of intake date allows us to determine whether service users meet the inclusion criteria of the EP program. Dx and DUP survey allow us to verify that service user has a psychosis-related diagnosis.* |
| Design-time | 17 | The data entry tool does not perform rounding or truncation of entries that might result in precision-loss | *Beehive does not truncate entries. This has been tested & corrected from alpha/beta versions of application.* |
| Design-time | 18 | Proxy responses for factual questions (such as employment status) are allowed in order to maximize completeness | *We have multiple respondents report on legal interactions, medication side-effects, social functioning, role functioning, and symptoms to maximize completeness of data.* |
| Design-time | 19 | Extract/transform/load software for batch upload of data from other sources such as assay results should flag impossible and implausible values | *In Beehive, the only data upload possible is for service user registration in batches. Each of these fields includes validation and impossible/implausible values will show an error and not be uploaded.* |
| Design-time | 20 | Internationalization is undertaken in a robust manner, and translation and cultural adaption of concepts (e.g., assessment tools) follows best practice | *All Beehive localization is completed with a certified translation (including back-translation). The Beehive EULA video is completely translated (including all on-screen text and the voice over). We have consulted with native speakers with an understanding of psychosis and/or serious mental illness to support with localization of the EULA video. Whenever possible, we have included Spanish-speaking individuals in our qualitative research as we have designed the core assessment battery and EULA video.* |
| Design-time | 21 | Data collection methods are documented in study manuals that are sufficiently detailed to ensure the same procedures are followed each time. | *We control for as much as we can through Beehive rules. For everything else, we provide the same training to all sites and make asynchronous training materials available to them. They have consistent support offered to them through their EPI-CAL point person.* |
| Design-time | 22 | All personnel responsible for entering data receive training and testing on how to complete the CRF. | *EPI-CAL staff conduct site trainings with all sites. This includes workflow meetings which operationalize how this process will be implemented in each program. Point persons offer ongoing support including re-training and booster trainings. Information is available for asynchronous learning in the resource guide and in recordings of site trainings. We provide training on the assessments which require training (COMPASS, GFS/GFR, Trauma Assessments).* |
| Design-time | 23 | The CRF/eCRF are easy to use and include a detailed description of the data collection guidelines and how to complete each field in the form. They are pilot tested in a rigorous pre-specified and documented process until reliability and validity are demonstrated | *We provide training on the assessments which require training (COMPASS, GFS/GFR, Trauma Assessments). Other parts of the CRF do not require training outside of the general Beehive trainings. Pilot testing on surveys was conducted with UCD students with mental illness. Alpha testing of application was conducted by UCD & UCSD EPI-CAL team. Beta testing was conducted with 3 EP CSC program pilot sites.* |
| Design-time | 24 | Data collectors are tested and provided with feedback regarding the accuracy of their performance across all relevant study domains. | *Reliability is assessed after COMPASS & GFS/GFR trainings. All other data collection does not require training outside of the training users should receive as part of their position at the EP program.* |
| Run-Time | 25 | Automated variable transformations are documented and tested before implementation and if modified | *Yes, and original variable is retained to allow for cross checking.* |
| Training and Testing | 26 | A team of data-curation experts are involved with pre-specified initial and ongoing testing for quality assurance | *We test data reports as part of regular testing of the application. Further, we are using data every week to track enrollment progress. Additionally, submissions to ENDCC have served as a guidepost for ensuring/checking data quality. Finally, we have an internal team for monitoring data for completeness and abnormalities.* |
| Run-time | 27 | There is a centralized monitoring of the completeness and consistency of information during data collection | *Dashboard visualizations and reviews of regular data downloads allow for monitoring of completeness and consistency of the data.* |
| Run-Time | 28 | Individual data elements should be checked for missingness. This should be done against pre-specified skip-logic/missingness masks. This should be performed throughout the study data acquisition period to give accurate 'real time ' feedback on completion status | *We code missing vs. skipped data so we can assess for completion. Dashboard shows visualizations of survey completion. Survey completion on an individual level is also visualized in Beehive. Survey missingness is monitored on a regular basis by point persons and data team.* |
| Run-Time | 29 | Systematic and timely measures are in place to assure ongoing data accuracy. | *We monitor survey completion and enrollment on a weekly basis.* |
| Design-time | 30 | Data collection that requires specific content expertise is carried out by trained and/or certified investigators. | *Beehive records the respondent ID of surveys (such as symptom assessment or GFS/GFR) and anyone gathering this data is required to complete training & reliability* |
| Design-time | 31 | Assessors are blinded to treatment allocation or predictor variables where appropriate and such blinding is explicitly recorded | N/A – *no treatment allocation* |
| Run-Time | 32 | Source data validation procedures are in place to check for agreement between the original data and the information recorded in the database. | *N/A -- data entered into Beehive is considered source data* |
| Run-time | 33 | Reliability checks have been performed on variables that are critical to research hypotheses, to ensure that information from multiple sources is consistent. | *We are monitoring these variables on a weekly basis. We also collect data for primary aims from 3 sources (service user, clinician, & PSP)* |
| Run-Time | 34 | The score of tests is checked. Scoring is performed automatically where possible. | *All scoring is performed automatically.* |
| Run-Time | 35 | Data irregularities are reported back to data collectors in a systematic and timely process. There is a standard operating procedure for data irregularities to be reported back to the data collectors and for documentation of the resolution of the issue. | *We monitor survey completion on a weekly basis. If discovered, irregularities will be reported to sites within the week.* |
| Design-time | 36 | There is a clear audit chain for any data processing that takes place after entry, and this should have a mechanism for version control if it changes. | *Any back-end changes (e.g., survey baseline date) must be approved by EPI-CAL staff and are documented in a ticketing system and in the secure research drive.* |
| Post-collection | 37 | The version lock-down of the database for data entry is clearly specified | *Survey data is locked after submission. Beehive provides a change log for any changes to service user registration information.* |
| Post-collection | 38 | A plan for ongoing curation and version control is specified | *Survey version is a variable available in survey reports. While the log of changes is not available within Beehive, EPI-CAL staff keep a log in box drive of significant changes made to surveys. Through Beehive, only the most current version of the survey is available to complete at any given time.* |
| Post-collection | 39 | A comprehensive data dictionary is available for end users | *Data dictionary is available for the research team, ENDCC, and all EP program users.* |
| Run-time | 40 | Known/emergent issues with the data dictionary are documented and reported in an accessible manner. | *Comments field for each variable. Also have a field for each variable of start/end date of data collection which helps to log variables which are planned and not yet implemented.* |
| Design-time | 41 | Data are provided in a form that is unambiguous to researchers | *Study form fields relationships are clearly identified in the data dictionary* |
| Design-time | 42 | For physiological data the methods of measurement and units are defined for all sites | *Units clearly specified in eCRF* |
| Design-time | 43 | Imaging acquisition techniques are standardized | *N/A* |
| Design-time | 44 | Biospecimen preparation techniques are standardized | *N/A* |
| Design-time | 45 | Biospecimen assay accuracy, precision, repeatability, detection limits, quantitation limits, linearity, and range are defined. Normal ranges are determined for each assay. | *N/A* |
| Design-time | 46 | There is automated entry of the results of biospecimen samples | *N/A* |
