# Supplementary material for: The California collaborative network to promote data driven care and improve outcomes in early psychosis (EPI-CAL) project: rationale, background, design and methodology
Source: BMC Psychiatry. 2024 Nov 14;24:800. doi: 10.1186/s12888-024-06245-6 (PMC11566177; doi:10.1186/s12888-024-06245-6)
Supplement: Supplementary file 2 — Supplementary Material 2 [file 12888_2024_6245_MOESM2_ESM.docx]

# Supplementary Table 1: EPI-CAL EP Program Detail

| Program* | Type | Clinical Population | Age | Max DUP (in months) | Service Length (in months) | Insurance(s) serviced | Other inclusion/exclusion criteria |
| --- | --- | --- | --- | --- | --- | --- | --- |
| UC, Davis EDAPT | University | SZ, affective psychosis, CHR, OSPD, substance induced psychosis | 12-40 | 24 | 19-24 | In-network Commercial Insurance, Service user self-pay | n/a |
| EMB aSPIRE SOAR Clinic (Sonoma) | CMHC. Other mental health agency | SZ, affective psychosis, CHR. Exclude substance induced psychosis | 12-30, (Kaiser, 12-25) | 24 | 19-24 | Medi-Cal, Limited Commercial Insurance (e.g., Kaiser) | Must be able to identify 1 support person. Sonoma County resident. |
| The Help Group | Other mental health agency. Non-profit | SZ, CHR, affective psychosis. Exclude substance induced psychosis | 12-25 | 24 | 12-18 | Medi-Cal | No active substance use. Must reside in Los Angeles County Service Planning Area 7 |
| Institute for Multicultural Counseling & Education Services (Koreatown) | CMHC | SZ, CHR, affective psychosis, substance induced psychosis | 12-25 | n/a | 19-24 | Medi-Cal | Must reside in Los Angeles County service planning area 4 or 6 |
| Institute for Multicultural Counseling & Education Services (West Covina) | CMHC | SZ, CHR, affective psychosis. Exclude substance induced psychosis | 12-25 | n/a | 19-24 | Medi-Cal | Must reside in Los Angeles County service planning area 3 |
| Aldea SOAR, Napa | CMHC | SZ, affective psychosis, CHR, OSPD. Exclude substance induced psychosis | 8-30 | 24 | 19-24 | Medi-Cal | Napa County resident |
| OC Center for Resiliency Education Wellness (OC CREW) | Other mental health agency | SZ, OSPD. Exclude substance induced, CHR, affective psychosis | 12-25 | 24 | 12-18 | Medi-Cal | Orange County resident |
| UC, Davis SacEDAPT | CMHC, Hospital. Academic Institution/University | SZ, affective psychosis, CHR, OSPD. | 12-30 | 24 | 19-24 | Medi-Cal, Uninsured | Sacramento County resident |
| Pathways Kickstart San Diego | CMHC | SZ, affective psychosis, CHR, OSPD. Exclude substance induced psychosis | 10-25 | 12 | 19-24 | Medi-Cal | San Diego County resident; Exclude intensive trauma work or behavioral issues |
| San Fernando Valley Community Mental Health Clinic | Other mental health agency | SZ, CHR, affective psychosis. Exclude substance induced psychosis | 12-25 | 12 | 12-18 | Medi-Cal, Uninsured | Must reside in Los Angeles County service planning area 2 |
| San Mateo Felton BEAM | CMHC | CHR, affective psychosis, substance induced psychosis only. Exclude brief psychotic episode | 14-34 | 24 | 19-24 | Medi-Cal, Commercial insurance | San Mateo County resident |
| San Mateo Felton reMIND | CMHC | SZ, affective psychosis, CHR, OSPD, substance induced. Exclude brief psychotic episode | 14-35 | 24 | 19-24 | Medi-Cal, Commercial Insurance. | San Mateo County resident |
| Aldea  SOAR, Solano | CMHC, Other mental health agency | SZ, affective psychosis, CHR, OSPD. Exclude substance induced psychosis | 14-25 | 24 | 19-24 | Medi-Cal, Uninsured, Limited Commercial Insurance (e.g., Kaiser) | Solano County resident (except Kaiser service users) |
| INSPIRE Clinic at Stanford University | Academic Institution/University. | SZ, affective psychosis, CHR, OSPD. Exclude substance induced psychosis | 16-65 | n/a | no limit | Commercial insurance (exclude Kaiser), Service user self-pay | n/a |
| LIFE Path | CMHC. Other mental health agency. | SZ, affective psychosis, CHR. Exclude substance induced psychosis | 14-25 | 12 | 12-18 | Medi-Cal | Stanislaus County resident |
| UC, Los Angeles Aftercare Research Program | Academic Institution/University | SZ. Exclude substance induced psychosis, CHR, affective psychosis | 18-45 | 24 | 19-24 | Only Grant funded. Academic funds | Need to speak English well enough to validly complete program/research measures |
| UC, Los Angeles CAPPS Program | Hospital. Academic Institution/University. | CHR (include mood), OSPD. Exclude FEP, substance induced psychosis, affective psychosis | 12-30 | n/a | 19-24 | Only Grants/ Gift funds/donors | Exclude significant substance use in last 3 months |
| UC, San Diego CARE Program | Hospital. Academic Institution/University. | SZ, affective psychosis, CHR, OSPD, substance induced psychosis | 14-35 | 60 | 37-42 | Commercial (must have contract with UCSD), Service user self-pay | n/a |
| UC, San Francisco PATH Program | Academic Institution/University | SZ, affective, CHR, OSPD, substance induced psychosis | 0-35 | Any | 19-24 | Commercial insurance | n/a |
| The Whole Child | CMHC, Other mental health agency | SZ, affective psychosis, CHR, substance induced psychosis | 12-25 | n/a | 19-24 | Medi-Cal | Must reside in Los Angeles County Service Planning Area 5, High acuity usually referred elsewhere |

*Additional programs are at various stages of the onboarding process into EPI-CAL, and specific program eligibility criteria is collected overtime as Beehive implementation takes place.

DUP = Duration of Untreated Psychosis, IQ = Intelligence Quotient, SZ = Schizophrenia spectrum disorders, CHR = Clinical High Risk, CMHC= Community Mental Health Center, OSPD = Other specified psychotic disorder, CAPPS = Center for Assessment and Prevention of Prodromal States, CARE = Cognitive Assessment and Risk Evaluation, EDAPT = Early Diagnosis and Preventative Treatment, SOAR = Supportive Outreach and Access to Resources, EMB = Elizabeth Morgan Brown, IMCES = Institute for Multicultural Counseling and Education Services, LIFE = Lasting Independence, Family Empowerment, BEAM = Bipolar Disorder Early Assessment and Management
